# Clinical Presentation and Targeted Genetic Evaluation of Familial Isolated Pituitary Adenomas: A Single-Center Case Series

**DOI:** 10.3390/diagnostics16213443

**Published:** 2026-10-22

**Authors:** Cristiana Călin, Iulia Florentina Burcea, Ramona Dobre, Cristina Căpățînă, Șerban Radian, Ionela Baciu, Elena Emanuela Braha, Cătălina Poiană

**Affiliations:** 1 Department of Endocrinology, Carol Davila University of Medicine and Pharmacy, 020021 Bucharest, Romania; iulia.burcea@umfcd.ro (I.F.B.); ramona.dobre@umfcd.ro (R.D.); cristina.capatina@yahoo.com (C.C.); serban.radian@gmail.com (Ș.R.); ionelafilip@yahoo.com (I.B.); endoparhon@gmail.com (C.P.); 2 First Endocrinology Department, C.I. Parhon National Institute of Endocrinology, 011863 Bucharest, Romania; 3 Department of Research, C.I. Parhon National Institute of Endocrinology, 011863 Bucharest, Romania; elenabraha@yahoo.com

**Keywords:** familial isolated pituitary adenomas, pituitary neuroendocrine tumors, acromegaly, AIP, genetic testing, nonfunctioning pituitary neuroendocrine tumors

## Abstract

**Background:** Familial isolated pituitary adenomas (FIPA) are characterized by considerable clinical and genetic heterogeneity. Although germline pathogenic variants in *AIP* account for a subset of cases, most FIPA families remain genetically unexplained. We aimed to describe the clinical characteristics, tumor phenotype, treatment outcomes, and targeted genetic evaluation of FIPA families from a tertiary endocrine referral center. **Methods:** We retrospectively identified FIPA families among patients with pituitary neuroendocrine tumors (PitNETs) evaluated at a tertiary endocrine referral center between 2019 and 2024. FIPA was defined as PitNETs occurring in at least two related individuals without clinical features suggestive of syndromic endocrine disease. Clinical, radiological, histopathological, and genetic data were analyzed. **Results:** Among 1068 patients with PitNETs, seven FIPA families were retrospectively identified (0.66% of the source cohort), comprising 20 affected individuals. Index cases included four women and three men, with a mean age at diagnosis of 37.1 ± 17.3 years. Somatotroph PitNETs predominated among index cases (5/7), whereas nonfunctioning PitNETs were more frequent among affected relatives (9/13). Six of seven index tumors were macroadenomas, and cavernous sinus invasion was present in two. No pathogenic or likely pathogenic variants in *AIP* or *MEN1* were identified by Sanger sequencing, and multiplex ligation-dependent probe amplification (MLPA) detected no copy number alterations involving *AIP*, *MEN1*, or *CDKN1B*. **Conclusions:** This series highlights the clinical heterogeneity of FIPA and the differing tumor spectrum between index cases and affected relatives. The absence of pathogenic variants identified by targeted genetic testing further illustrates the genetic heterogeneity of familial pituitary tumor predisposition.

## 1. Introduction

Although most pituitary neuroendocrine tumors (PitNETs) occur sporadically, a small proportion arise in a familial context. Familial isolated pituitary adenomas (FIPA) are defined as the occurrence of PitNETs in two or more related individuals within the same family in the absence of other hereditary endocrine syndromes such as multiple endocrine neoplasia type 1 (MEN1) or Carney complex [1,2,3]. Although the acronym FIPA has been retained because it is well established in the literature, the term PitNET is used throughout this manuscript in accordance with the current World Health Organization (WHO) classification [4,5]. FIPA accounts for approximately 2% of all pituitary tumors and represents the most common form of non-syndromic familial pituitary tumor predisposition [3,6]. Clinically, FIPA is characterized by marked heterogeneity in tumor subtype, age at diagnosis, and disease behavior [1,2,3].

Germline pathogenic variants in the aryl hydrocarbon receptor-interacting protein (*AIP*) gene represent the most frequent and best-characterized genetic cause of FIPA and were the first genetic alterations identified in association with familial PitNETs [3,6,7,8]. *AIP* functions as a tumor suppressor gene involved in intracellular pathways regulating cellular proliferation. *AIP*-associated PitNETs are predominantly somatotroph tumors and typically present at a younger age, with larger and more invasive tumors and a reduced response to first-generation somatostatin receptor ligands compared with sporadic PitNETs [3,9]. Genetic testing for *AIP* pathogenic variants is therefore recommended in patients with familial PitNETs and selected patients with young-onset disease, particularly those presenting with somatotroph or lactotroph macroadenomas at a young age [6,10,11,12,13,14]. Identification of an *AIP* pathogenic variant has important implications for genetic counseling and targeted screening of at-risk relatives.

Although *AIP* represents the best-established genetic cause of FIPA, the genetic background of familial pituitary tumor predisposition is heterogeneous. Pathogenic variants in genes associated with syndromic or other hereditary forms of pituitary tumor predisposition, including *MEN1*, *CDKN1B*, *PRKAR1A*, and *GPR101*, may also be identified in patients presenting with familial or early-onset PitNETs [12,13,15]. Nevertheless, most FIPA families remain without an identifiable genetic cause, suggesting that additional genetic or epigenetic mechanisms may contribute to disease susceptibility [6,12,13,14].

In this context, the present study aimed to characterize the clinical presentation, tumor phenotype, treatment outcomes, and results of targeted genetic evaluation in a single-center case series of FIPA families.

## 2. Materials and Methods

### 2.1. Study Design and Patient Selection

**Figure 1:**
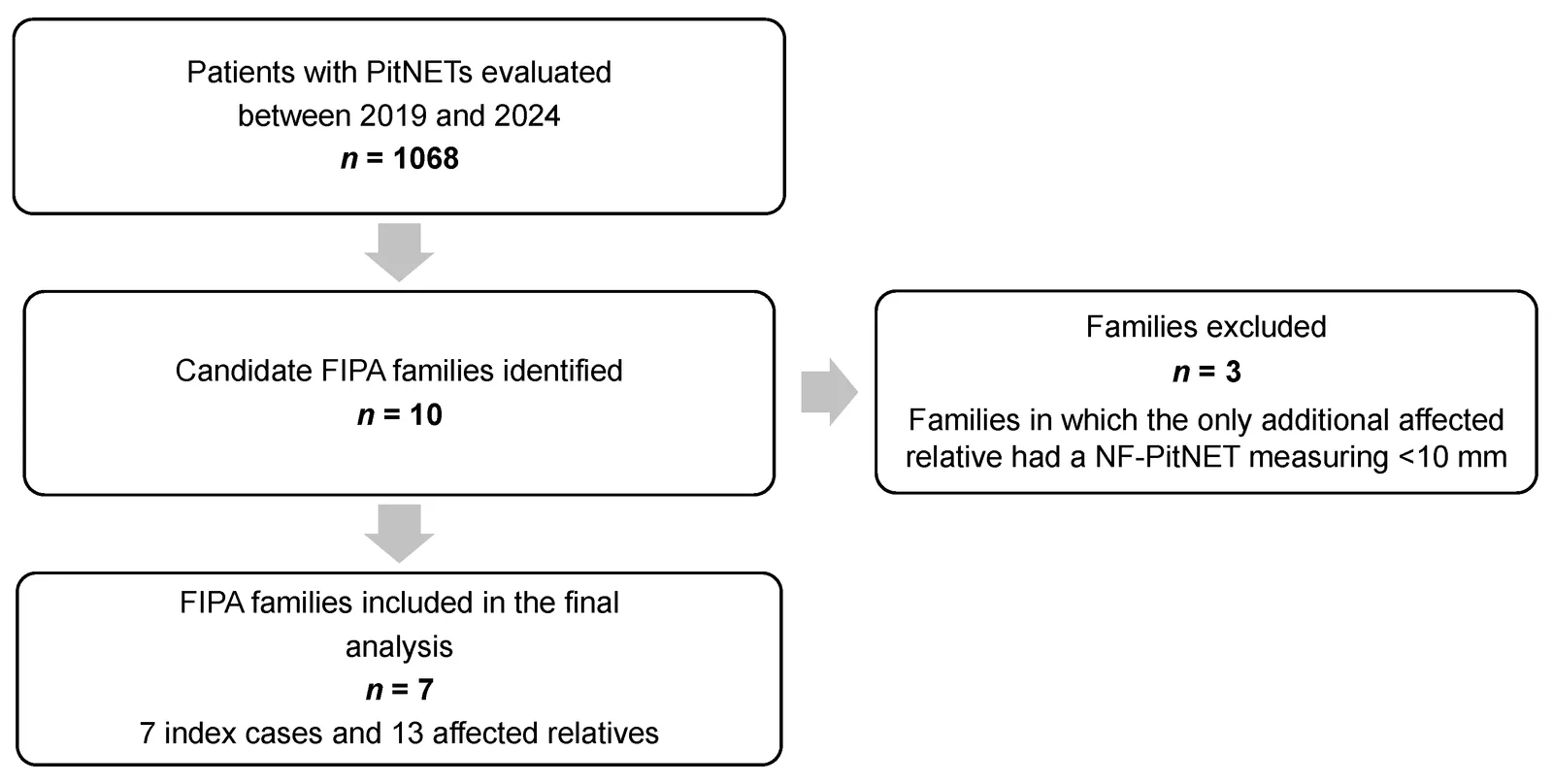
Flowchart illustrating the identification and selection of families with familial isolated pituitary adenomas (FIPA) from the source cohort. PitNET, pituitary neuroendocrine tumor; NF-PitNET, nonfunctioning pituitary neuroendocrine tumor.

We conducted a retrospective single-center study of patients with PitNETs evaluated at the C.I. Parhon National Institute of Endocrinology between January 2019 and December 2024. This period was used to identify the source cohort and ascertain eligible FIPA families. Clinical information preceding 2019, including initial diagnoses and previous treatments, was retrieved retrospectively from available medical records. The final cutoff date for clinical follow-up and data collection was 31 December 2024. All patients included in the source cohort were specifically questioned regarding a family history of diagnosed pituitary tumors. FIPA was defined as the presence of a documented PitNET in at least two biologically related individuals within the same family, in the absence of a recognized hereditary endocrine tumor syndrome [1,2,3,6]. Families in which the only additional affected relative had a nonfunctioning pituitary microadenoma (<10 mm) were excluded from the final analysis because of the possibility of an incidental pituitary lesion rather than true familial clustering. Three families met this exclusion criterion. The identification and selection of FIPA families from the source cohort are illustrated in Figure 1. The patient through whom familial clustering was identified within the source cohort was considered the index case. Affected relatives were identified retrospectively based on documented family history and were included only when the diagnosis of PitNET could be confirmed from available medical records, biochemical evaluation, and/or pituitary imaging. Relatives were not subjected to a uniform prospective biochemical and imaging screening protocol as part of the present study. All index cases underwent clinical and biochemical evaluation for manifestations suggestive of multiple endocrine neoplasia type 1 (MEN1), including assessment for primary hyperparathyroidism and measurement of chromogranin A, gastrin, insulin, serotonin, neuron-specific enolase, and urinary 5-hydroxyindoleacetic acid (5-HIAA). MEN1 genetic testing was performed in all index cases, as detailed below. The possibility of other hereditary endocrine tumor syndromes, including Carney complex, was assessed through clinical examination and review of the available medical and family histories. A standardized syndrome-specific screening protocol was not applied to affected relatives, whose evaluation relied on available retrospective clinical records.

### 2.2. Clinical, Radiological, and Treatment Outcome Assessment

Clinical data included demographic characteristics, hormonal evaluation, imaging findings, treatment history, and follow-up duration. Acromegaly was diagnosed based on clinical features, elevated age-adjusted insulin-like growth factor 1 (IGF-1) levels, and assessment of growth hormone (GH) secretion according to international consensus recommendations [16]. Prolactinomas were diagnosed in patients with persistent hyperprolactinemia and a pituitary lesion on magnetic resonance imaging (MRI), after exclusion of secondary causes of hyperprolactinemia, including medication-related hyperprolactinemia, primary hypothyroidism, renal impairment, and pituitary stalk compression [17]. Nonfunctioning PitNETs were defined as pituitary tumors without clinical or biochemical evidence of hormone hypersecretion [5]. Postoperative biochemical remission in patients with acromegaly was determined from the endocrine assessment documented in the medical records, generally performed 3–6 months after surgery, and was defined by normalization of age-adjusted serum IGF-1 levels without ongoing medical therapy. Patients without postoperative biochemical remission were considered to have persistent disease. Among patients requiring medical treatment, biochemical control was defined as normalization of age-adjusted serum IGF-1 levels, whereas persistently elevated IGF-1 levels indicated biochemically active disease. In patients with prolactinomas receiving dopamine agonist therapy, biochemical control was defined as normalization of serum prolactin levels. Radiological stability was defined as the absence of documented tumor growth or new tumor lesions on serial pituitary MRI examinations during follow-up. Disease status was determined according to the last available biochemical and radiological assessments within the study period. Tumor size was assessed by pituitary MRI, with macroadenomas defined as tumors ≥10 mm in maximum diameter [18]. Cavernous sinus invasion was defined as a Knosp grade > 2 (grades 3–4) [19]. Treatment modalities and clinical and biochemical outcomes during follow-up were retrieved from the medical records.

### 2.3. Histopathological Evaluation

For index cases who underwent pituitary surgery, histopathological data were retrieved from the corresponding pathology reports. Tumors were evaluated using hematoxylin and eosin staining and immunohistochemistry for anterior pituitary hormones, including GH, prolactin (PRL), adrenocorticotropic hormone (ACTH), thyroid-stimulating hormone (TSH), follicle-stimulating hormone (FSH), and luteinizing hormone (LH). In somatotroph PitNETs, the granulation pattern was assessed using cytokeratin immunohistochemistry (CAM5.2 or AE1/AE3), allowing classification as densely granulated, sparsely granulated, or mixed. The Ki-67 proliferation index was also recorded. Immunohistochemical assessment of pituitary lineage-specific transcription factors (PIT1, TPIT, and SF1) was not routinely performed during the study period and was therefore not systematically available for retrospective tumor classification according to the current WHO criteria [4,5,20,21,22].

### 2.4. Genetic Analysis

All seven index patients underwent targeted germline genetic testing after providing written informed consent. Genomic DNA was extracted from peripheral blood leukocytes using the Wizard^®^ Genomic DNA Purification Kit (Promega, Madison, WI, USA), and DNA concentration was assessed using a Qubit dsDNA HS Assay Kit (Thermo Fisher Scientific, Waltham, MA, USA). Sanger sequencing was performed following polymerase chain reaction (PCR) amplification of all six *AIP* exons and *MEN1* coding exons 2–10, including adjacent intronic regions for *MEN1*. Reference transcripts NM_003977.4 (*AIP*) and NM_001370259.2 (*MEN1*) were used for sequence annotation, with the GRCh38 (hg38) human genome assembly serving as the genomic reference. Sequencing reactions were performed using the GenomeLab DTCS QuickStart Kit (Beckman Coulter, Indianapolis, IN, USA) according to an in-house protocol, and products were analyzed using the CEQ 8000 Genetic Analysis System (Beckman Coulter, Indianapolis, IN, USA). Sequence analysis included visual inspection of chromatograms using DNA Baser Sequence Assembler v4 (Heracle BioSoft, Mioveni, Romania) and comparison with reference sequences. Copy-number analysis was performed using the SALSA MLPA Probemix P244-D1 AIP-MEN1-*CDKN1B* (MRC-Holland, Amsterdam, The Netherlands) to investigate deletions and duplications involving *AIP*, *MEN1*, and *CDKN1B*. All seven index patients were tested by *AIP* Sanger sequencing (7/7), *MEN1* Sanger sequencing (7/7), and multiplex ligation-dependent probe amplification (MLPA) (7/7). Sequence variants were classified according to the American College of Medical Genetics and Genomics and Association for Molecular Pathology (ACMG/AMP) recommendations as pathogenic (class 5), likely pathogenic (class 4), variants of uncertain significance (class 3), likely benign (class 2), or benign (class 1) [23]. Sequence-level analysis of *CDKN1B* and broader next-generation sequencing panels for hereditary pituitary tumor predisposition were not performed. Affected relatives did not undergo systematic genetic testing as part of this retrospective study.

### 2.5. Statistical Analysis

Given the retrospective design and small sample size, statistical analysis was limited to descriptive methods. Categorical variables were summarized as absolute frequencies and percentages, while continuous variables were expressed as mean ± standard deviation (SD), median, and range. Descriptive statistics were calculated using Microsoft Excel for Mac, version 16.47 (Microsoft Corporation, Redmond, WA, USA). Analyses were based on available data for each variable, without imputation of missing values. Where applicable, percentages were calculated using the number of individuals with available information as the denominator. No inferential statistical comparisons were performed between index cases and affected relatives because of differences in ascertainment and the non-independence of individuals belonging to the same families.

## 3. Results

### 3.1. Identification and Characteristics of FIPA Families

Among 1068 patients with PitNETs evaluated at our center between 2019 and 2024, seven families fulfilled the study criteria for FIPA, corresponding to 0.66% of the source cohort. These families comprised 20 affected individuals, including seven index cases and 13 affected relatives. Three additional families were excluded because the only additional affected relative had a nonfunctioning pituitary microadenoma (<10 mm), according to the predefined exclusion criterion. The clinical characteristics and tumor phenotypes of the seven included families are summarized in Table 1.

### 3.2. Clinical Characteristics of Index Cases

The seven index cases included four women and three men, with a mean age at PitNET diagnosis of 37.1 ± 17.3 years (median, 35 years; range, 17–60 years). Five patients presented with acromegaly, one with a prolactinoma, and one with an NF-PitNET. The mean maximum tumor diameter at diagnosis was 20.1 ± 9.8 mm (median, 21 mm; range, 6–36 mm). Six patients had macroadenomas and one had a microadenoma. Cavernous sinus invasion, defined as a Knosp grade > 2, was present in two of the seven index cases. None of the seven index cases had documented clinical or biochemical manifestations suggestive of MEN1, including primary hyperparathyroidism or a functioning gastroenteropancreatic neuroendocrine tumor. No clinical features suggestive of Carney complex were documented. The individual clinical characteristics, tumor features, treatment modalities, and follow-up duration are summarized in Table 2.

### 3.3. Treatment and Follow-Up

All index cases underwent transsphenoidal surgery as primary treatment, except for the patient with a prolactinoma, who was managed medically with cabergoline.

Preoperative pituitary hormone deficiencies were present in two index cases. The patients from Families 1 and 6 had central hypothyroidism and hypogonadotropic hypogonadism before surgery, which persisted postoperatively. The index case from Family 2 presented with optic chiasm compression and visual impairment before surgery.

Among the five index cases with acromegaly, four achieved biochemical remission following surgery without requiring additional medical therapy. In Family 2, biochemical remission was achieved following the third transsphenoidal intervention. The index case from Family 1 had persistent biochemically active disease after surgery and required multimodal medical treatment. Combination therapy with octreotide long-acting release (LAR), pegvisomant, and cabergoline failed to achieve biochemical control. Treatment was therefore switched to pasireotide LAR in combination with cabergoline; response to the current regimen had not yet been assessed at the end of the study period.

The index case with an NF-PitNET underwent surgical treatment and remained clinically and radiologically stable during follow-up, without evidence of tumor progression. The patient with a prolactinoma was managed medically with cabergoline and achieved biochemical control.

Postoperative complications were limited. The index case from Family 2 developed panhypopituitarism after surgery and had persistent visual impairment related to preoperative optic chiasm compression. The patient from Family 3 developed transient postoperative hyponatremia, which subsequently resolved. No postoperative complications were documented in the remaining surgically treated index cases. The mean follow-up duration for the index cases was 9.6 ± 5.3 years (median, 10 years; range, 1–15 years).

### 3.4. Histopathological Findings

Histopathological evaluation was available for all five index cases with surgically treated somatotroph PitNETs and confirmed somatotroph tumors by positive GH immunostaining. No hormone co-expression was identified. Cytokeratin immunohistochemistry (CAM5.2 or AE1/AE3) showed a sparsely granulated pattern in the tumor of the index case from Family 1, whereas the tumors of the index cases from Families 2–5 were densely granulated.

Histopathological evaluation of the NF-PitNET index case (Family 6) showed negative immunostaining for all assessed anterior pituitary hormones. As lineage-specific transcription factor immunohistochemistry was unavailable, further classification according to the current WHO criteria could not be established.

The Ki-67 proliferation index was <3% in all evaluated cases.

### 3.5. Affected Relatives

A total of 13 affected relatives were identified across the seven families. Nonfunctioning PitNETs were the most frequent tumor subtype, occurring in nine relatives (69.2%), while acromegaly and prolactinomas were each identified in two relatives (15.4%). Eight relatives had macroadenomas and five had microadenomas, with a mean maximum tumor diameter of 13.2 ± 7.9 mm (median, 12 mm; range, 4–27 mm). The individual clinical characteristics, treatment, and clinical course of all 13 affected relatives are summarized in Supplementary Table S1.

**Figure 2:**
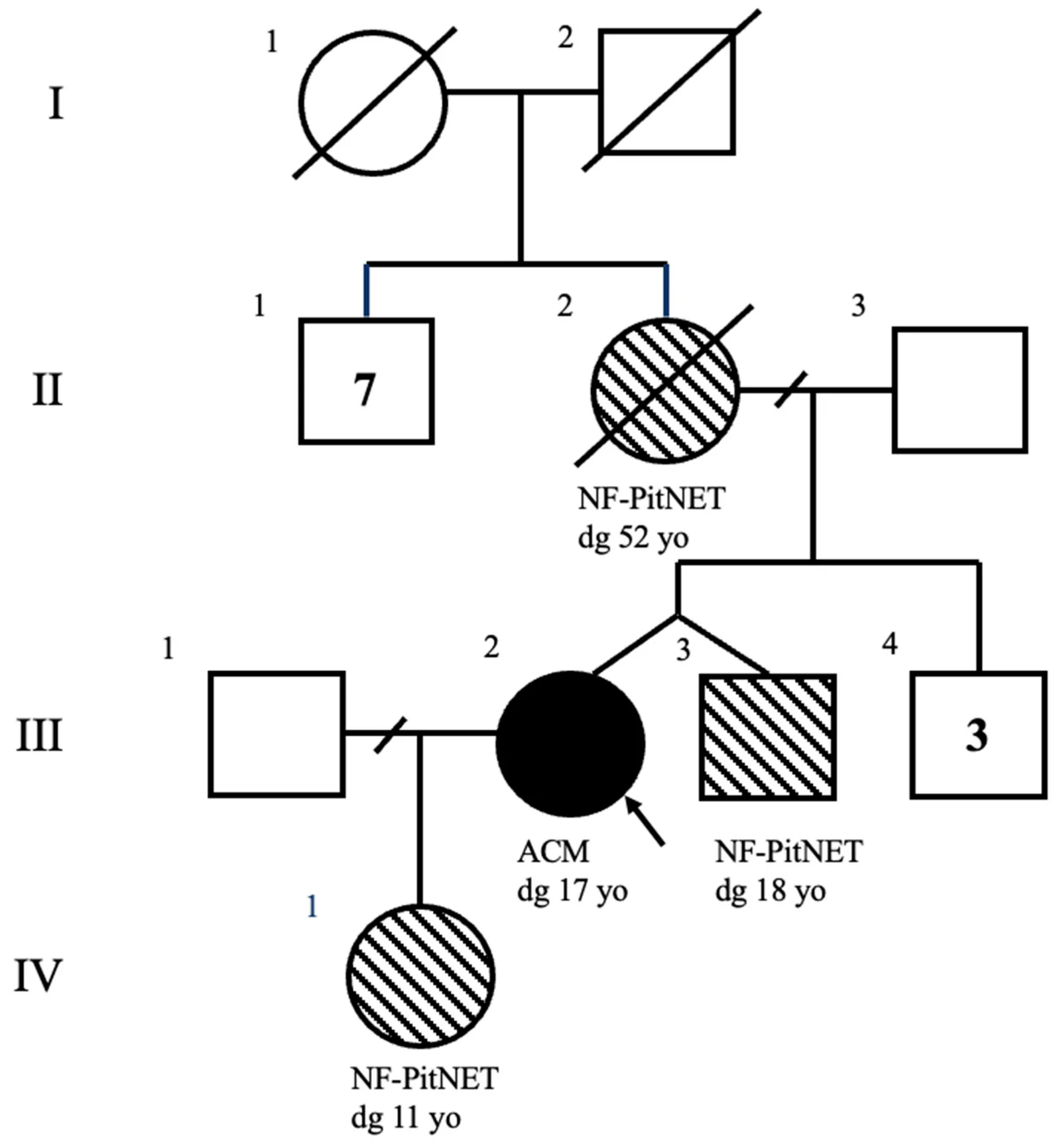
Pedigree of Family 1 (F1), comprising the index case with acromegaly and three affected relatives with nonfunctioning PitNETs. Note: Squares indicate males and circles indicate females. Black-filled symbols indicate acromegaly, whereas striped symbols indicate NF-PitNETs. Open symbols denote unaffected family members. Deceased individuals are indicated by a diagonal slash. A slash through a relationship line indicates divorce. Numbers within symbols indicate additional unaffected siblings not individually represented. The index case is indicated by an arrow.

The pedigree of Family 1 is presented in Figure 2, while the pedigrees of the remaining six families are provided in the Supplementary Material (Figures S1–S6). Family 1 comprised four affected individuals across three generations: the index case with acromegaly and three relatives with NF-PitNETs.

### 3.6. Targeted Genetic Evaluation

Targeted germline genetic evaluation was performed in all seven index cases. Sanger sequencing of *AIP* and *MEN1* identified no sequence variants across ACMG/AMP classes 1–5, including benign, likely benign, variants of uncertain significance, likely pathogenic, or pathogenic variants. MLPA analysis identified no copy-number alterations involving *AIP*, *MEN1*, or *CDKN1B*.

## 4. Discussion

In this single-center series, seven FIPA families comprising 20 affected individuals were identified among 1068 patients with PitNETs evaluated during the study period. The series illustrates substantial intrafamilial and interfamilial phenotypic heterogeneity, with acromegaly predominating among index cases and NF-PitNETs representing the most frequent phenotype among affected relatives. Most index cases presented with macroadenomas, while targeted germline genetic evaluation did not identify sequence variants in *AIP* or *MEN1* or copy-number alterations involving *AIP*, *MEN1*, or *CDKN1B*.

The seven FIPA families represented 0.66% of the source cohort, a proportion lower than the approximately 2% reported in the literature [3,6]. However, this difference should be interpreted in light of the study-specific ascertainment and inclusion criteria. Although all patients in the source cohort were specifically questioned regarding a family history of diagnosed pituitary tumors, relatives were not subjected to systematic biochemical or pituitary imaging screening. Consequently, asymptomatic or previously unrecognized PitNETs among family members may have remained undetected, potentially leading to an underestimation of familial clustering. Furthermore, three additional candidate families were excluded because the only other affected relative had a nonfunctioning pituitary microadenoma (<10 mm). Although this conservative criterion was intended to minimize the risk of misclassifying incidental pituitary lesions as familial disease, it may also have excluded genuine FIPA families.

Comparisons with contemporary studies should also account for differences in ascertainment strategies. In the multicenter study by Ertorer et al. [24], 45 FIPA families were identified, of which 20 were selected for detailed clinical and genetic characterization. Unlike our study, which identified familial clustering within a defined cohort of patients with PitNETs, this study focused on previously identified familial cases. Furthermore, Marques et al. [10] demonstrated the clinical benefits of genetic testing and systematic screening of at-risk relatives, highlighting the importance of family-based ascertainment in identifying otherwise unrecognized pituitary tumors. These differences in recruitment strategies, diagnostic ascertainment, and inclusion criteria limit direct comparisons of FIPA frequency across studies. Therefore, the observed proportion of 0.66% should be regarded as the frequency of families meeting our predefined criteria within the source cohort rather than an estimate of the true prevalence of FIPA.

A notable finding was the different distribution of PitNET phenotypes between index cases and affected relatives. Five of the seven index cases presented with acromegaly, whereas NF-PitNETs accounted for 69.2% of tumors among affected relatives. However, this difference should be interpreted cautiously because of potential ascertainment and detection biases. Index cases were identified among patients evaluated at a tertiary endocrine referral center, where clinically apparent functioning tumors, particularly those causing acromegaly, may be more readily recognized. In contrast, affected relatives were identified retrospectively through documented family history and available clinical, biochemical, or imaging records, rather than through a standardized family screening protocol. Consequently, the observed phenotype distribution among relatives may reflect differences in diagnostic pathways and availability of medical documentation rather than the true distribution of PitNET subtypes within FIPA families. In addition, asymptomatic or previously undiagnosed pituitary tumors may have remained unidentified. Therefore, comparisons between index cases and affected relatives are descriptive and should not be interpreted as evidence of a genuine difference in phenotype prevalence between these groups.

Considerable intrafamilial heterogeneity was also observed. Five of the seven families included more than one PitNET phenotype, while only two families were phenotypically homogeneous. Family 1 provides a representative example, with the index case presenting with acromegaly and three affected relatives across successive generations presenting with NF-PitNETs. Such phenotypic variability is well recognized in FIPA and illustrates that the tumor phenotype of the index case does not necessarily predict the phenotype occurring in other affected family members [1,2,3].

Most index tumors in our series were macroadenomas, and two showed cavernous sinus invasion according to the predefined Knosp criterion. Younger age at presentation, larger tumor size, invasiveness, and reduced responsiveness to first-generation somatostatin receptor ligands (SRLs) have been particularly associated with *AIP*-related somatotroph PitNETs [3,6,9,13]. However, no *AIP* sequence or copy-number alterations were identified in our index cases; therefore, the clinical characteristics observed in this small series should not be interpreted as an *AIP*-associated phenotype. Of interest, the index case from Family 1, who presented at 17 years of age and required multimodal treatment for persistent acromegaly, had a sparsely granulated somatotroph PitNET, whereas the remaining four somatotroph tumors were densely granulated. Sparsely granulated somatotroph PitNETs have been associated with reduced responsiveness to first-generation SRLs, which may have contributed to the treatment course observed in this patient [21,22,25]. The combination of early-onset acromegaly, a challenging treatment course, and familial clustering across three generations raises the possibility of an underlying hereditary predisposition despite negative targeted *AIP* testing. However, these clinical findings are not specific to *AIP*-related disease, and the molecular basis of pituitary tumor predisposition in this family remains unresolved. Nevertheless, observations from a single family do not allow broader conclusions regarding genotype–phenotype associations or treatment response in FIPA.

No sequence variants across ACMG/AMP classes 1–5 were identified in *AIP* or *MEN1*, and MLPA detected no copy-number alterations involving *AIP*, *MEN1*, or *CDKN1B*. Our findings are consistent with previous FIPA cohorts in which most families had no identifiable *AIP* alterations. Daly et al. reported *AIP* alterations in 11 of 73 families (15.1%) [8], while Hernández-Ramírez et al. identified pathogenic or likely pathogenic *AIP* variants in 37 of 216 families (17.1%) [26]. More recently, Ertorer et al. reported *AIP* variants in three of 20 families (15%) using whole-exome sequencing, alongside additional variants in syndromic and candidate genes [24]. Together, these findings highlight the substantial genetic heterogeneity of FIPA and demonstrate that a considerable proportion of families remain without an established molecular diagnosis. Nevertheless, differences in genetic testing strategies and the small size of our cohort limit direct comparisons.

The genetic findings must also be interpreted in light of the testing strategy. Although Sanger sequencing assessed the coding regions and exon-intron boundaries of *AIP* and *MEN1*, and MLPA allowed detection of large deletions and duplications involving *AIP*, *MEN1*, and *CDKN1B*, sequence-level analysis of *CDKN1B* was not performed. Moreover, broader next-generation sequencing panels for hereditary pituitary tumor predisposition were not available as part of the diagnostic strategy used during the study period. Consequently, pathogenic sequence variants in *CDKN1B*, alterations involving other genes implicated in pituitary tumor predisposition, including *GPR101*, *PRKAR1A*, *SDHx*, *MAX*, and *DICER1*, and genetic mechanisms not detectable using the employed methods cannot be excluded. Although these genes are associated with distinct hereditary or syndromic contexts, their inclusion in broader genetic evaluations may be relevant in selected patients, depending on the clinical phenotype and family history. The absence of variants identified by the targeted approach should therefore not be interpreted as excluding an underlying hereditary predisposition.

Genetic testing was performed in the index cases but was not systematically extended to affected relatives as part of this retrospective study. In families with an identified pathogenic germline variant, cascade testing of relatives is particularly informative because it allows identification of variant carriers who may benefit from clinical surveillance, including individuals who have not yet developed a PitNET because of incomplete penetrance [6,10,11]. In the present series, however, no pathogenic or likely pathogenic variant was identified in any index case, precluding variant-directed cascade testing. Nevertheless, the absence of an identifiable variant does not eliminate familial risk, and clinical awareness among relatives remains relevant in genetically unexplained FIPA families.

Several limitations should be acknowledged. First, this was a retrospective single-center study including only seven families, which limits the generalizability of the findings and precludes meaningful genotype–phenotype analyses. Second, although family history of diagnosed pituitary tumors was specifically assessed in the source cohort, relatives were not subjected to a standardized prospective biochemical and imaging screening protocol; therefore, additional asymptomatic affected relatives may have remained unidentified. Furthermore, although clinical and biochemical evaluation and targeted *MEN1* genetic testing did not identify evidence of MEN1 among the index cases, these investigations cannot definitively exclude all forms of syndromic predisposition. The absence of systematic syndrome-specific screening among affected relatives and of a comprehensive evaluation for Carney complex further limits the certainty with which an underlying syndromic predisposition can be excluded. Third, the genetic evaluation was targeted and did not include sequence-level analysis of *CDKN1B* or broader next-generation sequencing. Fourth, affected relatives did not undergo systematic germline genetic testing. Finally, lineage-specific transcription factor immunohistochemistry (PIT1, TPIT, and SF1) was not routinely available during the study period, limiting retrospective histopathological classification according to the current WHO framework.

Despite these limitations, this series provides a detailed clinical, radiological, histopathological, and targeted genetic characterization of FIPA families evaluated at a tertiary endocrine referral center. The differing phenotype distribution between index cases and affected relatives, together with marked intrafamilial heterogeneity and the absence of pathogenic variants identified by targeted genetic testing, underscores the complexity of familial pituitary tumor predisposition. Careful assessment of family history remains important in patients with PitNETs, while advances in broader genomic approaches may help clarify the genetic basis of currently unexplained FIPA families.

## 5. Conclusions

This single-center case series highlights the marked clinical and phenotypic heterogeneity of FIPA, both between and within affected families. Acromegaly predominated among index cases, whereas NF-PitNETs were more frequently identified among affected relatives. No pathogenic variants were identified using the targeted genetic approach employed in this study, emphasizing that a substantial proportion of familial pituitary tumor predisposition remains genetically unexplained. Careful assessment of family history remains important in patients with PitNETs, while broader genetic approaches may contribute to elucidating the molecular basis of genetically unresolved FIPA families.

## Figures and Tables

**Table 1:** Distribution of PitNET phenotypes among the seven FIPA families.

Family	Index Case Phenotype	Affected Individuals, *n*	Acromegaly, *n*	Prolactinoma, *n*	NF-PitNET, *n*
F1	Acromegaly	4	1	0	3
F2	Acromegaly	3	1	0	2
F3	Acromegaly	2	1	0	1
F4	Acromegaly	5	2	0	3
F5	Acromegaly	2	2	0	0
F6	NF-PitNET	2	0	1	1
F7	Prolactinoma	2	0	2	0

Abbreviations: FIPA, familial isolated pituitary adenomas; NF-PitNET, nonfunctioning pituitary neuroendocrine tumor; PitNET, pituitary neuroendocrine tumor.

**Table 2:** Clinical characteristics, tumor features, treatment, and follow-up of the seven index cases.

Family	Clinical Phenotype	Age, *y*	Size, *mm*	Knosp	Surgeries, *n*	Status	Therapy	FU, *y*
F1	Acromegaly	17	22	2	2	Active	PAS + CAB	14
F2	Acromegaly	55	21	2	3	Remission	-	13
F3	Acromegaly	60	6	0	1	Remission	-	10
F4	Acromegaly	35	12	1	1	Remission	-	15
F5	Acromegaly	21	17	2	1	Remission	-	4
F6	NF-PitNET	48	36	3	2	Stable	-	10
F7	Prolactinoma	24	27	3	0	Controlled	CAB	1

Note: Age and maximum tumor diameter refer to values at diagnosis. Knosp grade was assessed on MRI at diagnosis. Disease status and medical therapy refer to the last available follow-up assessment within the study period. Follow-up duration was calculated from diagnosis to the last clinical evaluation. Abbreviations: CAB, cabergoline; FU, follow-up; NF-PitNET, nonfunctioning pituitary neuroendocrine tumor; PAS, pasireotide.

## Data Availability

The data supporting the findings of this study are available from the corresponding author upon reasonable request. The data are not publicly available due to privacy and ethical restrictions related to patient and family-level clinical and genetic information.

## Supplementary Materials

The following supporting information can be downloaded at https://www.mdpi.com/article/10.3390/diagnostics16213443/s1. Figure S1: Pedigree of Family 2 (F2); Figure S2: Pedigree of Family 3 (F3); Figure S3: Pedigree of Family 4 (F4); Figure S4: Pedigree of Family 5 (F5); Figure S5: Pedigree of Family 6 (F6); Figure S6: Pedigree of Family 7 (F7). Table S1. Individual clinical characteristics of the 13 affected relatives from seven familial isolated pituitary adenoma (FIPA) families.
