# Supplementary material for: Clinical Presentation and Targeted Genetic Evaluation of Familial Isolated Pituitary Adenomas: A Single-Center Case Series
Source: Diagnostics (Basel). 2026 Oct 22;16(21):3443. doi: 10.3390/diagnostics16213443 (PMC13761853; doi:10.3390/diagnostics16213443)

Supplementary Material

Supplementary Table

Supplementary Table S1. Individual clinical characteristics of the 13 affected relatives from seven familial isolated pituitary adenoma (FIPA) families.

**Family/ID**	**Kinship**	**Sex**	**Age (years)**	**PitNET type**	**Size (mm)**	**Treatment**	**Clinical course / FU**
**F1-R1**	Mother	F	52	**NF-PitNET**	12	None	Radiologically stable, deceased at 53 (CV cause), 1 year
**F1-R2**	Twin brother	M	18	**NF-PitNET**	13	None	Radiologically stable, 5 years
**F1-R3**	Daughter	F	11	**NF-PitNET**	12	None	Radiologically stable, 3 years
**F2-R1**	Mother	F	62	**NF-PitNET**	14	None	NA
**F2-R2**	Daughter	F	40	**NF-PitNET**	6	None	Radiologically stable, 5 years
**F3-R1**	Father	M	77	Invasive **NF-PitNET** with optic chiasm compression	27	None (inoperable)	Tumor progression; deceased at 83 (CV cause), 6 years
**F4-R1**	Father	M	51	**NF-PitNET**	7	None	Radiologically stable, 10 years
**F4-R2**	Paternal uncle	M	45	**ACM**	12	Transsphenoidal surgery	Biochemical remission; deceased at 48 (CV cause), 1 year
**F4-R3**	Son	M	18	**NF-PitNET**	5	None	Radiologically stable, 7 years
**F4-R4**	Daughter	F	13	**NF-PitNET**	4	None	Radiologically stable, 7 years
**F5-R1**	Sister	F	25	**ACM**	25	Transsphenoidal surgery, fg-SRL + CAB	Biochemically controlled under medical therapy, 9 years
**F6-R1**	Sister	F	32	**PRL**	9	CAB	Biochemically controlled, 12 years
**F7-R1**	Father	M	44	**PRL**	26	CAB	Biochemically controlled, 1 year

Abbreviations: ACM, acromegaly; CAB, cabergoline; CV, cardiovascular; F, female; fg-SRL, first-generation somatostatin receptor ligand; FU, follow-up; M, male; NA, not available; NF-PitNET, nonfunctioning pituitary neuroendocrine tumor; PRL, prolactinoma.

Note: Age and tumor size refer to the time of diagnosis. Tumor size represents the maximum tumor diameter. Follow-up duration is expressed in years. Clinical course reflects the last available evaluation.

Supplementary Figures

Legend for Supplementary Figures S1-S6. Squares indicate males and circles indicate females. Black-filled symbols indicate somatotroph PitNETs (acromegaly), striped symbols indicate nonfunctioning PitNETs, and dotted symbols indicate prolactinomas. Open symbols denote unaffected family members. A diagonal slash through a symbol indicates a deceased individual. A slash through the relationship line indicates divorce. A triangle indicates a stillbirth. Numbers within symbols indicate additional unaffected siblings not individually represented in the pedigree. The index case is indicated by an arrow.

Abbreviations: ACM, acromegaly; NF-PitNET, nonfunctioning pituitary neuroendocrine tumor; PRL, prolactinoma.

Supplementary Figure S1. Pedigree of Family 2.

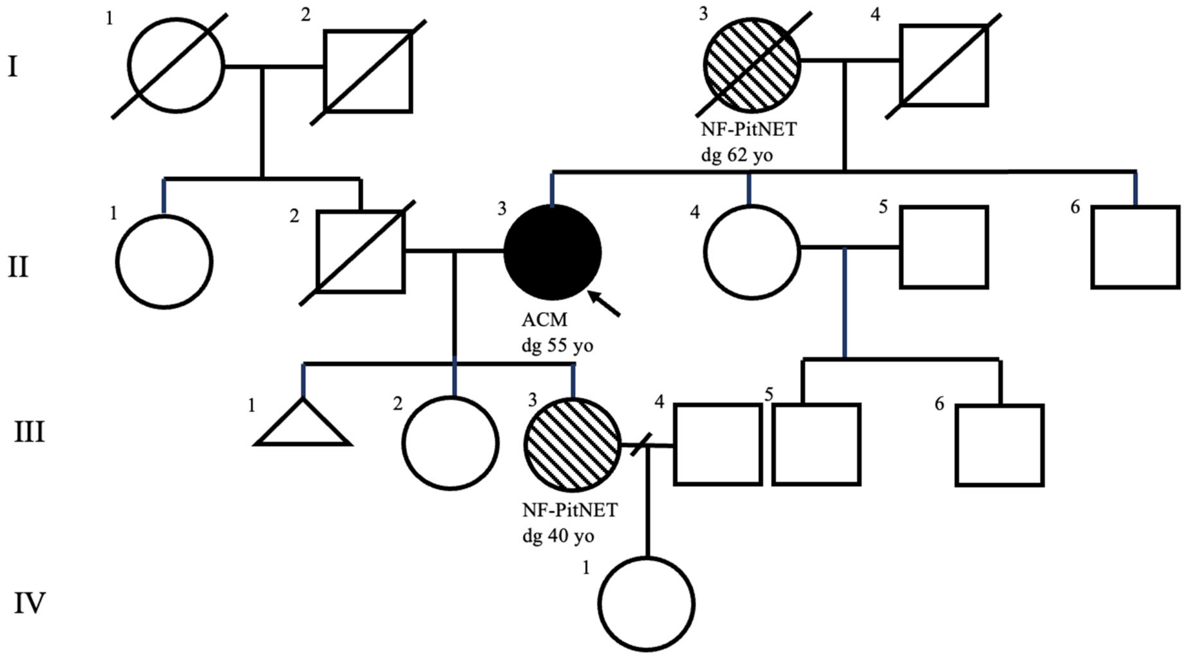

Supplementary Figure S2. Pedigree of Family 3.

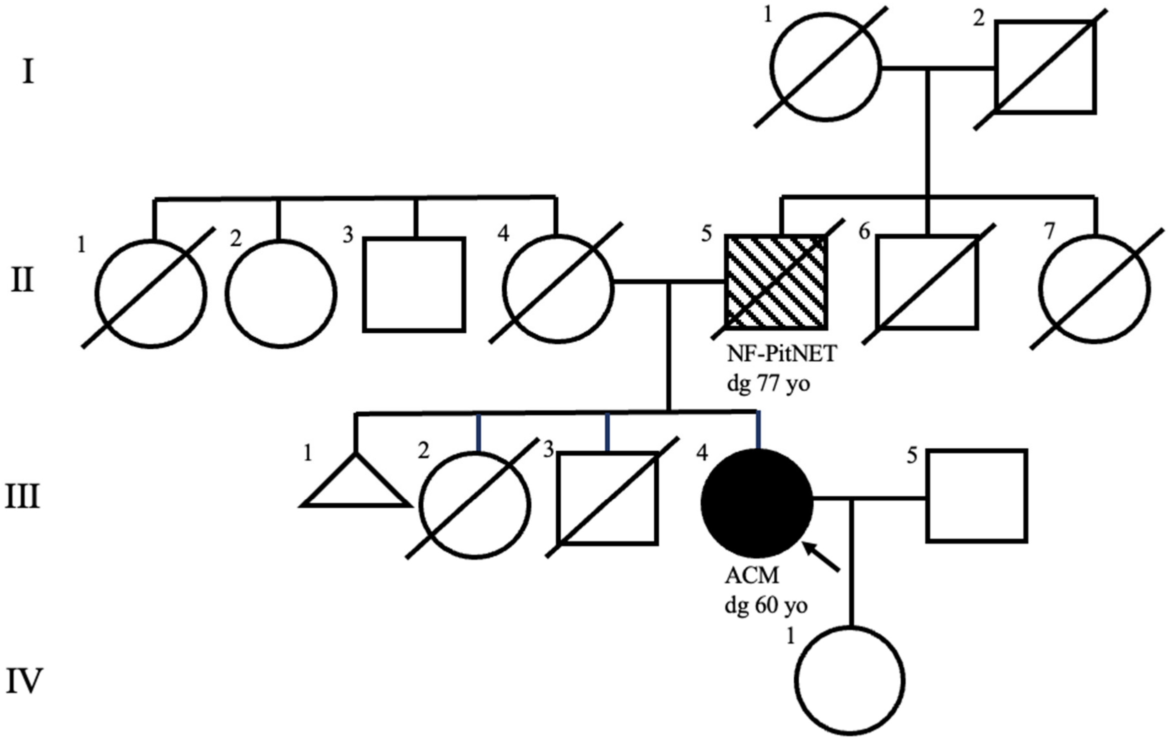

Supplementary Figure S3. Pedigree of Family 4.

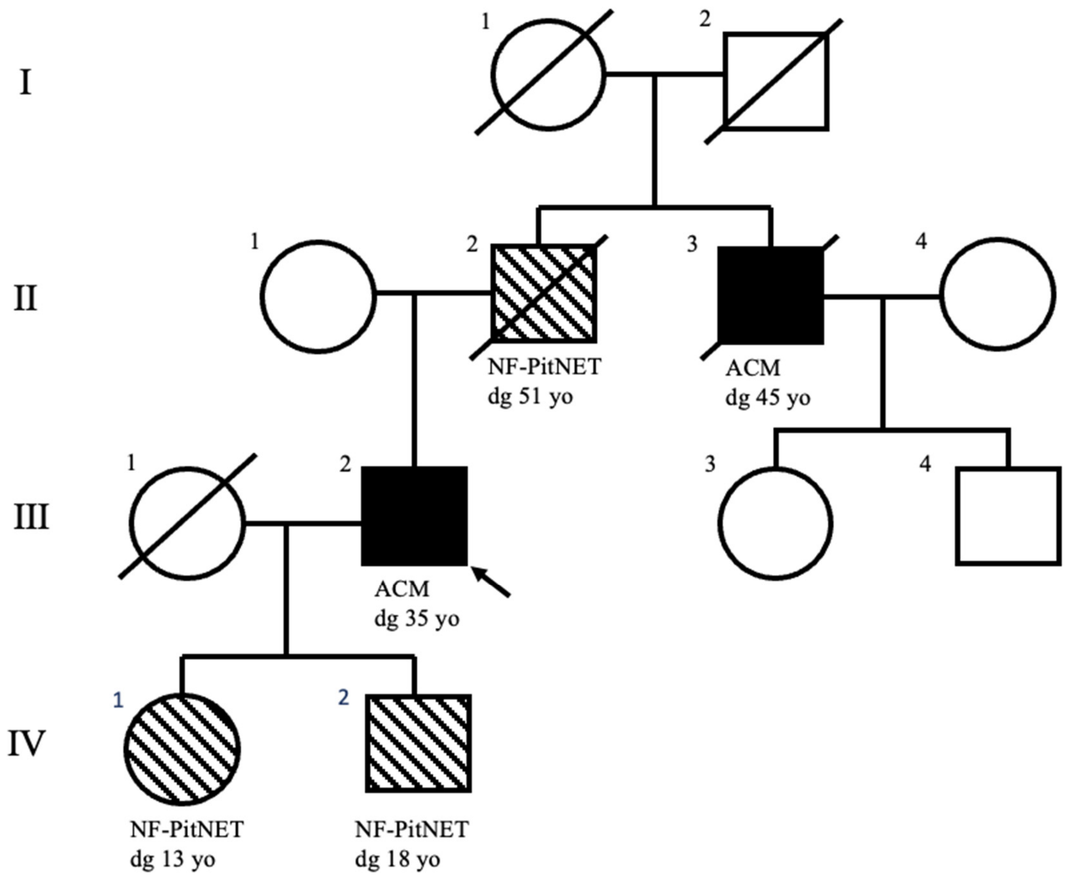

Supplementary Figure S4. Pedigree of Family 5.

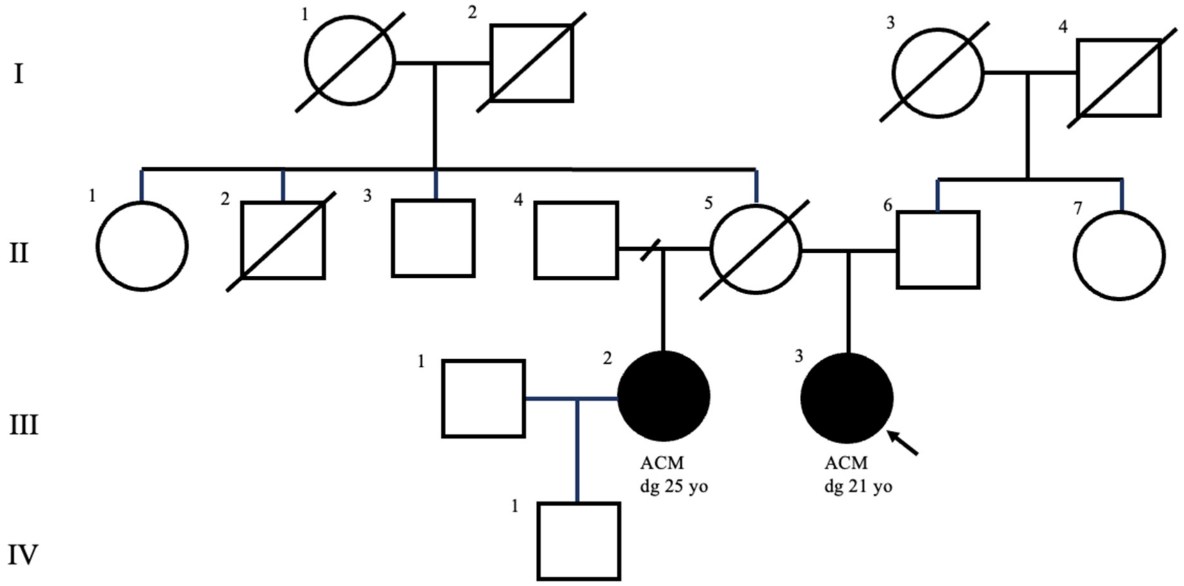

Supplementary Figure S5. Pedigree of Family 6.

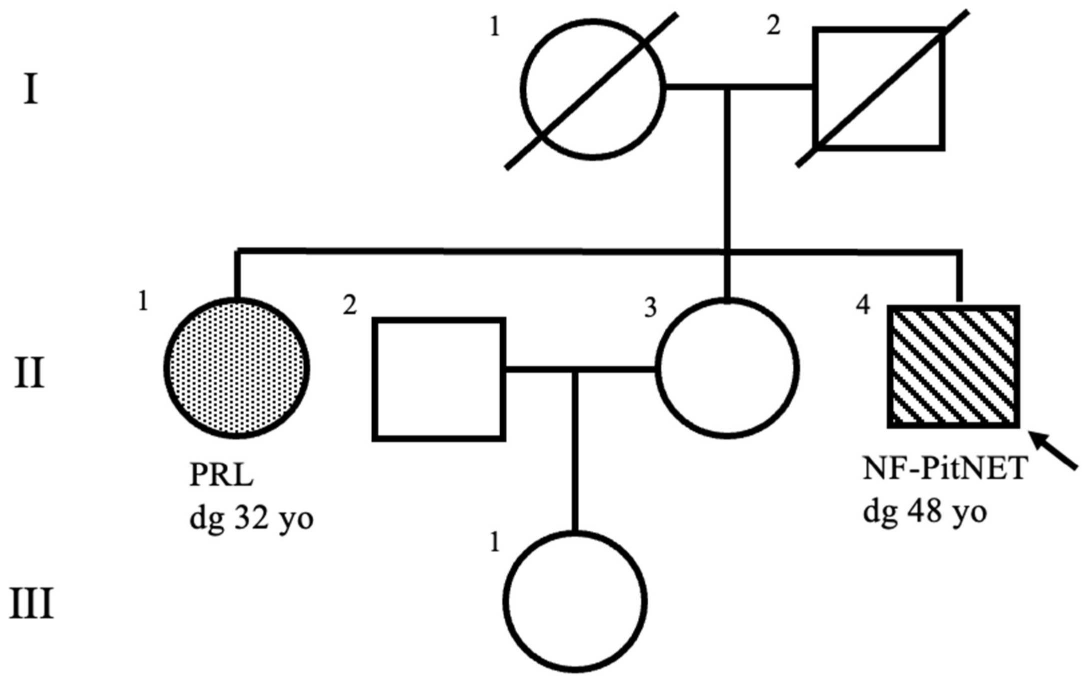

Supplementary Figure S6. Pedigree of Family 7.

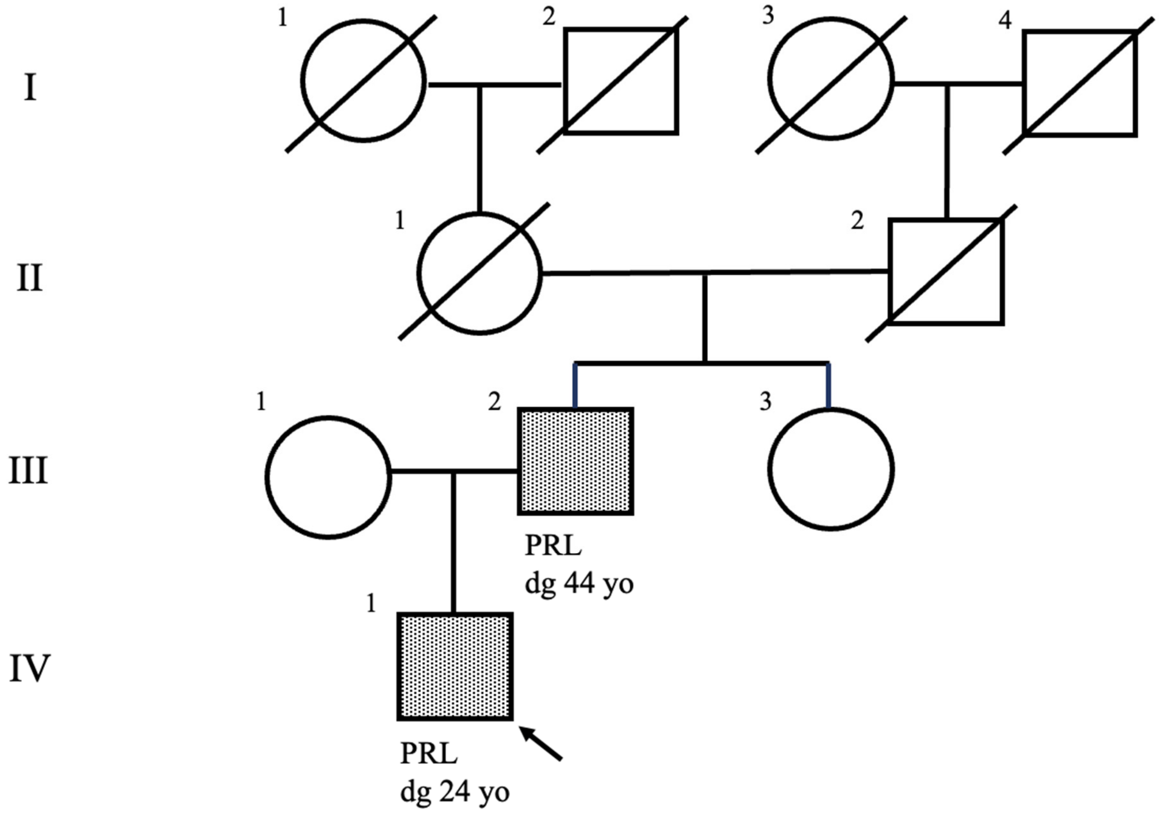

Supplement: Supplementary file 1 [file diagnostics-16-03443-s001.zip › diagnostics-4608526-supplementary.pdf]
